# Predictors of resilience in healthcare workers during the COVID-19 pandemic: a longitudinal study comparing the first and second waves

**DOI:** 10.1186/s40359-023-01077-7

**Published:** 2023-05-02

**Authors:** Irhomis Mendoza Bernal, David Sánchez-Teruel, María Auxiliadora Robles-Bello, Aziz Sarhani-Robles, Mariam Sarhani-Robles

**Affiliations:** 1 Psychology Department, University of Jaen, Campus Las Lagunillas, s/n, 23071 Jaén, Spain; 2 Faculty of Psychology, University of Granada, Granada, Spain; 3 Spanish Society of Suicidology, Madrid, Spain; 4 Medicine Faculty, University of Granda, Granada, Spain; 5 Medicine Faculty, Autonomous University of Barcelona, Barcelona, Spain

**Keywords:** Emotional intelligence, Self-efficacy, Optimism, Resilience, Healthcare workers, COVID-19, Protection factors, Risk factors

## Abstract

Few studies have investigated the effects of the pandemic caused by COVID-19 on health professionals, especially nurses, from the point of view of the protective factors of mental health. The aim of this study was to assess the level of resilience in healthcare workers, to determine whether there were differences between two moments of the pandemic. Applying a longitudinal study, participants (N = 590) from healthcare workers completed surveys in the first wave of the COVID-19 pandemic and the second wave. Socio-demographic and psychosocial variables such as resilience, emotional intelligence, optimism, self-efficacy, anxiety, and depression are used. There were differences between the two waves in all protective and risk variables except anxiety. In the first wave, there were three socio-demographic and psychosocial variables that explained 67.1% of the variance in resilience. In the first wave, three sociodemographic and psychosocial variables explained 67.1% of the variance in resilience in healthcare professionals. The enhancement of specific protective variables in healthcare professionals exposed to situations of high emotional stress can minimise the negative impact of the situation and promote more resilient responses in this professional group as a result.

## Introduction

The COVID-19 pandemic has strained the healthcare and economic systems of the vast majority of the world's countries [1, 2]. This crisis has had a major impact on the physical and emotional health of the population, due to the combination of social isolation imposed by health authorities and continued loss of life [3]. Health workers have also been badly affected due to a series of circumstances that have tested their emotional management, containment, and resilience [4]. Some of the circumstances included the overwhelming workload caused by lack of foresight in health systems, insufficient personal protective equipment, continued risk of infection, emotional pressure, ethical and moral dilemmas, and fear of infecting their own family members [5], although these same situations also permitted the development of individual and collective resources and strengths which were perhaps unknown until now [6]. Despite that, few studies have been conducted on which variables promoted higher levels of resilience during the COVID-19 pandemic in these healthcare professionals.

Resilience was originally considered as the human capacity to flexibly face adverse situations and overcome them [7]. The American Psychological Association [8] defined it as a process of adequate adaptation to adversity or trauma or even to significant sources of stress. It is currently understood as a complex process considering protective factors at the cognitive, emotional, and behavioural levels [9] that produce a positive adaptation to a hostile environment associated with personal growth [10, 11]. Thus, in specific adverse situations, the interaction of specific protective factors promotes adaptive processes that can culminate in resilient rather than psychopathological outcomes [12]. Resilient processes are complex and people who experience an adverse situation find themselves within a network made up of many elements that sustain them [13]. These protective elements are situationally specific [14, 15], which drives the need to determine which of them modulate high levels of resilience in health professionals exposed to situations of varying adversity [16].

In Spain, several studies have been conducted exploring mental health problems during the first wave in Spanish nursing students, showing an increase in fear, anxiety, depression, and sleep problems [17, 18]. Similar data were found in healthcare professionals during the early period of the pandemic, reporting a high incidence of anxiety, depression, and posttraumatic stress among participants [19]. The impact of COVID-19 was not equal among all healthcare professionals as being a woman, a nurse, and working shift increased the incidence of anxiety, depression, and posttraumatic stress among participants in cross-sectional studies [20, 21]. Variables such as resilience have also been studied in nursing students and professionals in cross-sectional studies where they found lower symptomatology of anxiety, depression, posttraumatic stress, and burnout [19, 21]. In Spain, there are few studies looking for a relationship between resilience, protective variables associated with it and sociodemographic variables in nursing professionals from a longitudinal perspective, although there is one study in which nursing students became professionals during the pandemic [22].

In Spain, the COVID-19 pandemic period can be divided into two periods. The first, which lasted from mid-March to the end of June 2020, was characterized by the entire population in Spain being confined to their homes and the collapse of the healthcare system, and the second, or so-called "new normal", from June 2020 onwards, with the relaxation of those previous restrictions. This led to the first wave of the pandemic (March 2020) being followed by a second wave (October 2020). In March 2020, hospitals were overflowing with COVID-19 patients and the lack of protective equipment meant that Spanish healthcare workers suffered the highest rate of infections in the world [23]. Spaniards were getting sick and there was a lack of professionals to care for them, ICU beds in hospitals and ventilators that could save their lives [24] and the most vulnerable, especially elderly people in nursing homes, could not always be cared for [24], also, healthcare workers (medical and nursing staff) became the profession most affected by contagions. The ICUs of many hospitals could not cope and despite the implementation of field hospitals, a study estimated that half of the deaths in the first wave were the result of hospital overcrowding [25]. The peak of infection was reached at the end of March and the peak of deaths in the first days of April. In just 24 h, on April 1, Spain registered 950 deaths [25]. On June 21, three months later, Spain came out of the State of Alarm. The so-called de-escalation process—the progressive withdrawal of confinement measures and mobility restrictions—had begun in early May and on June 21, when all of Spain entered the "new normal" the 14-day cumulative incidence was eight cases per 100,000 inhabitants [22]. This was the situation in Spain during the year 2020.

In the second wave, with incidences exceeding 500 14-day cases per 100,000 inhabitants in the second wave in October and November and without strict confinements, the downward speed of the curve did not achieve as much speed as expected and overlapped with the third wave, which began to shoot up in early January 2021 after a Christmas of meetings and national mobility [26].

The aim of this study was to assess the level of resilience in Spanish healthcare professionals and to determine whether there were differences between the initial part of the COVID-19 pandemic (March 2020) and several months later (October 2020). We also attempted to analyse which socio-demographic and psychosocial variables were more predictive of high levels of resilience in these healthcare professionals. Therefore, it is hypothesized that there will be significant differences between both temporal moments experienced during the pandemic with respect to resilience, although those protective variables of mental health measures (dispositional optimism, self-efficacy, and emotional intelligence), of nursing professionals, will have a direct and significant relationship with resilience and an inverse and significant relationship with risk variables measured (anxiety and depression).

## Method

### Participants

The sample consisted of 590 healthcare professionals (nursing) (376–63.7% women) with a mean age of 38.12 years (SD = 7.23) assessed during the first and second waves of the COVID-19 pandemic. Inclusion criteria were: Working in a hospital or health centre and agreeing to participate in the study and signing their informed consent online. There were 288 participants (202–52.0% women and 186–47.9% men with a mean age of 39.12 years; SD = 8.36) in the first wave (March 2020), while in the second wave (October 2020) there were 302 participants (174–57.6% women and 128–42.4% men with a mean age of 37.28 years; SD = 6.49). There were no significant differences in gender and age between the samples at the two time points (*p* > 0.05). The descriptive socio-demographic data are shown in Table 1.

**Table 1:** Sociodemographic data of nursing

	Total N(%)	First Wave n(%)	Second Wave n(%)	t	η^2^	Power
Marital status				0.34^ns^	0.45	0.72
Single	111 (18.81)	52 (18.06)	59 (19.53)			
Partner/married	245 (41.53)	123 (42.71)	122 (40.40)			
Separated/Divorced	169 (28.64)	81 (28.12)	88 (29.14)			
Widowed	65 (11.02)	32 (11.11)	33 (10.93%)			
Lives with children				0.65^ns^	0.36	0.69
No	217 (36.78)	104 (36.11)	113 (37.42)			
Yes	373 (63.22)	184 (63.89)	189 (62.58)			
Years of experience in healthcare				0.92^ ns^	0.71	0.66
1–6	93 (15.76)	39 (13.54)	54 (17.88)			
7–12	144 (24.41)	65 (22.57)	78 (25.83)			
13–18	198 (33.56)	102 (35.42)	97 (32.12)			
More than 18	155 (26.27)	82 (28.47)	73 (24.17)			
In contact with COVID-19 + patients				1.32^**^	0.98	0.83
No	98 (16.61)	82 (28.47)	16 (5.3)			
Yes	492 (83.39)	206 (71.53)	286 (94.70)			
Type of dwelling				0.46^ns^	0.22	0.59
Flat of less than 59 square meters	46 (7.80)	24 (8.33)	23 (7.61)			
Flat between 60 and 99 square meters	101 (17.12)	42 (14.58)	59 (19.54)			
Flat of 100 square meters or more	164 (27.80)	85 (29.52)	78 (25.83)			
One-story house of 100 square meters	195 (33.05)	91 (31.60)	105 (34.77)			
Two-story house of 100 square meters	84 (14.23)	46 (15.97)	37 (12.25)			
	590 (100)	288 (100)	302 (100)	0.39^ ns^	0.61	0.94

First Wave = March 2020; Second Wave = October 2020; t = Student-T; * = *p* < 0.05; ** =* p*  <  0.01; ns = Not significant; η2 = eta square; Power = Power of contrast

### Measures

Socio-demographic data sheet: sex, age, civil status, cohabitation with children, years of professional healthcare experience, contact with COVID-19 + patients, and type of dwelling.

*Wong and Law’s Emotional Intelligence Scale (WLEIS)* [27], translated and adapted to Spanish by Vila and Pérez-González [28]. This scale is composed of 16 items and is based on the definition of Emotional Intelligence proposed by Mayer and Salovey, [29] consisting of four dimensions each with four items: (1) perception of one's own emotions (e.g., "Most of the time I can distinguish why I have certain feelings"); (2) perception of the emotions of others (e.g., "I am sensitive to the feelings and emotions of others"); (3) use of emotions (e.g., "I always tell myself that I am a competent person"); and (4) emotion regulation (e.g., "I am able to control my temper and handle difficulties rationally"). The response format uses a seven-point Likert-type scale (1 = completely disagree and 7 = completely agree), with higher scores indicating higher emotional intelligence. The authors in the original study [27], reported indices of internal consistency from 0.83 to 0.90. The participants completed the Spanish version of the scale, which has good validity and reliability in Spanish population [30]. In this study, Cronbach's alpha for the total was 0.96.

*Life Orientation Test-Revised (LOT-R)* [31] adapted to Spanish by Ferrando et al. [32]. This instrument uses a five-point Likert-type scale (0 = strongly disagree and 4 = strongly agree) and has 10 items: three statements on optimism (items 1, 4, and 10), three on pessimism (items 3, 7, and 9), and four distractor items (2, 5, 6, 8) whose scores are not part of the calculation. Only the items measuring optimism were used in this study. Cronbach's alpha for the Spanish adaptation was 0.70 for optimism and test–retest correlations were 0.68. In this study, Cronbach's alpha was 0.70.

*General Self-Efficacy Scale* (GSES) [33], translated into Spanish by Sanjuán et al. [34]. This scale evaluates beliefs of self-efficacy in certain life situations. It correlates positively with self-esteem, optimism, and job satisfaction and has negatively with anxiety, depression, and physical symptoms [35]. It consists of 10 four-point Likert-type items, where 1 is "not true" and 4 is "completely true". The total score is calculated by adding together all item scores, giving a total score ranging from 10 to 40. There are no cut-off points, the higher the score, the higher the overall perceived self-efficacy. The original version has adequate internal consistency (Cronbach's alpha between 0.76 and 0.90). The internal consistency of the Spanish version was 0.84 [36]. In this study, Cronbach's alpha for professionals was 0.94.

*Hospital Anxiety and Depression Scale (HADS)* by Zigmond and Snaith [37] adapted and validated in Spanish by Tejero et al. [38]. The version by Herrero et al. [39] was used in this study. The HADS assesses the intensity and frequency of anxiety and depressive symptoms in recent weeks in various types of samples (general and clinical). It has 14 items that are divided into two subscales, each with seven items (HADA: anxiety and HADD: depression) and uses a Likert-type scale with four response alternatives. The total score for each of the subscales ranges from 0 to 21 (where scores between 0 and 7 = no anxiety/depression, between 8 and 10 = doubtful or possible symptomatology, and scores above 10 indicate a clinical problem). The test–retest reliability, internal consistency, and validity indices are very good for both the clinical [38] and non-clinical [40] Spanish populations. Internal consistency is adequate for both subscales, always above 0.70 and in the vast majority of studies above 0.80, regardless of the sample evaluated (physical, psychiatric, or health problems). [41] In this study, Cronbach's alpha was 0.76.

The 10-item *Connor-Davidson Resilience Scale (CD-RISC10)* [42]) was translated and adapted to Spanish by Notario-Pacheco et al. [43]. It measures resilience from a perspective of the ability to adapt to adversity in order to tolerate experiences such as change, personal problems, illness, pressure, failure, and painful feelings (item examples: "are able to adapt to change", "Tend to bounce back after illness or difficulty" and "Can maintain concentration under pressure"). Each item is scored on a five-point Likert-type scale, from 0 (strongly disagree) to 4 (strongly agree), and the total score ranges from 0 to 40. In terms of psychometric properties, it has good internal consistency (alpha = 0.87) [44]. In this study, Cronbach's alpha was 0.90.

### Design study

This is a longitudinal study as data collection was conducted during two different waves of the COVID-19 pandemic: the first wave, covering March-April 2020, and the second, in October-November 2020.

### Procedure

During the data collection period in the first wave, the entire Spanish population was in confinement, due to the government declared state of alarm for dealing with the COVID-19 crisis, which was decreed in mid-March and lasted until the end of June. In the second data collection period, the Spanish population had returned to a "new normality" [45], although at the end of October another state of alarm was declared, although this time with less restrictive measures than before, consisting mainly of a curfew, a ban on travel between autonomous communities, and limitations on the number of non-cohabitants who could gather.

A snowball sampling method was used to send participants a link to a Google form containing the questionnaire. The link was distributed through social networks such as Facebook and Instagram, and email contacts were also used. Participants received no remuneration for participation in the study. Participants completed the questionnaires in Spanish through an online survey platform (Google Forms, licensed by the University of Jaen in Spain). In the first evaluation, data collection was online, which first included informed consent and express agreement to participate, without which the participant could not access the evaluation instruments. For the next evaluation, those who had previously agreed to participate in the longitudinal study were contacted by email. Participants were also informed that the data obtained would be confidential and would be treated in accordance with the relevant data protection legislation, EU Regulation 2016/679 of the European Parliament and of the Council of 27 April 2016, Organic Law 3/2018 of 5 December on the Protection of Personal Data and Guarantee of Digital Rights. The study was approved by the ethics committee of the University of Jaén (Spain) (code: JUL.22/5.LÍNEA; DIC.20/9.TFM) and followed the ethical guidelines of the Spanish Society of Psychology and the principles of the Declaration of Helsinki [46].

### Data analysis

Missing data accounted for less than 1% of all variables and was replaced using a multiple imputation method (SPSS) [47] (Graham, 2012). First, preliminary descriptive analyses were carried out for the two waves of the pandemic using Student's t-test. The relationships between resilience and protective variables (self-efficacy, dispositional optimism, and emotional intelligence) and risk variables (depression and anxiety) were analysed using Pearson's correlation coefficient. Finally, a stepwise multiple regression was performed on the socio-demographic, protective, and risk variables for each wave in order to determine which variables were more predictive of resilience. The level of statistical significance required for all tests was a minimum of *p* < 0.05. Statistical analysis of the data was performed using SPSS version 28.0 (IBM Corporation, 2021) and statistical power and effect sizes were determined using G*Power 3.1.9.7 [48].

## Results

There were significant differences in the results between the two time points in almost all psychosocial variables (protective and risk), except for anxiety (*p* > 0.05) (Table 2). All protective variables were lower in the second wave (W2), while risk variables increased (W2), except for anxiety levels, which were similar at both time points.

**Table 2:** Descriptive data for the sample of healthcare professionals

	W1				W2				t	η^2^	Power
	M(SD)	K–S	S(SE) (0.29)	K(SE) (0.57)	M(SD)	K–S	S(SE) (0.50)	K(SE) (0.98)			
Emotional intelligence	77.1 (5.7)	0.19	0.38	0.12	49.12 (4.3)	0.26	0.19	0.86	4.1*	0.37	0.43
Optimism	11.3 (1.5)	0.22	0.29	0.43	3.7 (2.2)	0.61	0.22	0.34	18.3**	0.22	0.77
Self-efficacy	36.2 (2.1)	0.31	0.71	0.83	16.7 (3.4)	0.94	0.71	0.56	21.4**	0.86	0.81
Anxiety	12.6 (3.8)	0.14	0.25	− 0.51	12.1 (4.2)	0.43	0.28	− 1.8	1.6^ ns^	0.14	0.56
Depression	5.4 (3.7)	0.71	0.75	− 0.08	25.6 (4.0)	0.81	1.36	1.99	26.7**	0.28	0.91
Resilience	30.4 (6.3)	0.54	− 0.75	0.68	18.5 (7.0)	0.65	− 0.27	− 1.38	21.9**	0.76	0.88

W1 = March 2020; W2 = October 2020; M = Mean; *SD* = Standard Deviation; K–S = Kolmogorov–Smirnov Test; S = Skewness; K = Kurtosis; SE = Standard error; S-W = Shapiro–Wilk test; t = Student-T; * =* p* < 0.05; ** =  *p* < 0.01; ns = Not significant; η2 = eta square; Power = Power of contrast

During both waves, there were significant positive relationships between resilience and all protective variables (optimism, self-efficacy, and emotional intelligence), along with a significant inverse relationship with anxiety and depression (Table 3). More specifically, in the first wave of the COVID-19 pandemic, the strongest positive relationship was between self-efficacy and resilience, while the strongest inverse correlation was between anxiety and resilience. In the second wave, optimism had the strongest positive relationship with resilience, whereas depression had the strongest inverse correlation.

**Table 3:** Correlation of Psychosocial Variables in Wave 1 and Wave 2

	Emotional intelligence	Optimism	Self-efficacy	Anxiety	Depression	Resilience
W1						
Emotional intelligence	1					
Optimism	0.46**	1				
Self-efficacy	0.48**	0.49**	1			
Anxiety	− 0.34**	− 0.32**	− 0.27**	1		
Depression	− 0.21**	− 0.24*	− 0.14*	0.59**	1	
Resilience	0.63**	0.50**	0.87**	− 0.77**	− 0.27**	1
W2						
Emotional intelligence	1					
Optimism	− 76**	1				
Self-efficacy	0.53**	0.75**	1			
Anxiety	− 0.58**	− 0.58**	− 0.43*	1		
Depression	− 0.44*	− 0.56**	− 0.44*	0.70**	1	
Resilience	0.61**	0.82**	0.74**	− 0.64**	− 0.88**	1

W1 = March 2020; W2 = October 2020; t = T-Student; * = *p* < 0.05; ** = *p* < 0.01; ns = Not significant

The results about which variables (socio-demographic and psychosocial) were most predictive of resilience in health professionals in March (W1) and October (W2) 2020 were obtained through multivariate regression analysis, with the adequacy of the data being analysed prior to that. First, the assumption of independence of errors (Durbin-Watson = 1–3) was met with a coefficient close to two (DW_w1_ = between 1.81 and 1.97; DW_w2_ = 1.75–1.98). In addition, the variance inflation factor (VIF) showed that there was no multicollinearity in the predictor variables according to the time points (VIF_w1_ = between 1 and 1.723; VIF_w2_ = 1–2.493).

The final proposed model (model 3) indicated that in March 2020 (W1), the independent variables explained 67.1% of the variance in resilience (R^2c^ = 0.671; F = 54.93; *p* < 0.01) in healthcare professionals (Table 4). The variables that best predicted a high level of resilience were having between 7 and 12 years of professional experience in healthcare, having a high level of self-efficacy, and to a lesser extent the use of emotions within emotional intelligence and being optimistic. However, the final model most predictive of a resilient response in health professionals in October 2020 (W2) was model 2, which explained 71.1% of the variance in resilience (R^2c^ = 0.711; F = 42.910; *p* < 0.01). More specifically, having between 7 and 12 years of professional health experience, having been in contact with positive COVID-19 patients, being optimistic, and to a lesser extent not perceiving the emotions of others. The effect sizes were large and statistical power was high.

**Table 4:** Multiple regression for (sociodemographic and psychosocial) variables predicting resilience in each wave

	*DW*	*R*^2c^	*F*	B	SE	*t*	β	CI (95%) β		1−β	ƒ^2^
								LL	UL		
**W1**											
*Model 1*	1.81	0.591	97.85**							0.66	0.75
Marital Status				1.12	2.57	1.23*	0.19	0.03	0.26		
Lives with children (yes)				0.39	0.08	2.45*	0.22	0.08	1.26		
Years’ experience (7–12)				0.63	0.42	4.23**	0.66	0.61	0.82		
Self-efficacy				0.91	0.23	9.90*	0.78	0.64	0.97		
*Model 2*	1.93	0.643	60.74**							0.73	0.81
Lives with children (yes)				1.29	0.50	0.12*	0.02	− 0.04	2.36		
Years’ experience (7–12)				0.16	0.11	5.31**	0.93	0.11	0.98		
Self-efficacy				0.35	0.74	7.70**	0.64	0.49	0.84		
Emotional intelligence (UOE)				0.54	0.83	3.21**	0.27	0.13	0.55		
*Model 3*	1.97	0.671	54.93**							0.99	4.91
Years (7–12)				1.98	0.73	4.39**	0.56	0.29	0.74		
Self-efficacy				0.87	0.12	5.97**	0.54	0.38	0.76		
Emotional intelligence (UOE)				0.54	0.73	2.71**	0.23	0.07	0.49		
Optimism				0.49	0.92	2.32*	0.20	0.04	0.51		
**W2**											
*Model 1*	1.75	0.615	50.31**							0.68	0.75
Marital Status				8.12	3.72	0.45*	0.21	0.11	0.52		
Lives with children (yes)				1.89	1.01	0.24*	0.74	0.69	1.11		
Years’ experience (7–12)				0.93	0.87	2.47*	0.91	0.48	1.99		
COVID-19 + (Yes)				0.29	0.01	4.28**	0.84	0.29	1.27		
Optimism				0.51	0.09	5.51**	0.78	0.50	1.11		
*Model 2*	1.98	0.711	42.10**							0.99	2.96
Years’ experience (7–12)				1.23	0.12	4.23**	0.51	0.32	0.78		
COVID-19 + (Yes)				0.94	0.16	4.11*	0.21	0.08	0.43		
Optimism				0.54	0.81	5.81**	1.16	0.76	1.62		
Emotional intelligence (OEA)				0.98	0.05	− 2.44*	− 0.49	− 0.11	− 0.74		

W1 = March 2020; W2 = October 2020; Years’ experience = Years of experience in healthcare; Emotional intelligence (UOE) = Emotional Intelligence (sub-dimension of emotions); Emotional intelligence (OEA) = Emotional Intelligence (sub-dimension perception of the emotions of others); COVID-19 +  = In contact with positive COVID-19 patients; DW = Durbin-Watson Test; R^2c^ = corrected coefficient of determination; F = test statistic (ANOVA); *p < 0.05 ** p < 0.01; ns = not significant; SE = standard error; t = test statistic for predictor variables; β = regression result or beta equation; CI = confidence intervals; LL = lower limit; UL = upper limit; 1−β = statistical power; ƒ^2^ = effect size

## Discussion

This study aimed to assess the levels of resilience in healthcare professionals (nursing) in order to determine whether there were differences between the initial period of the COVID-19 pandemic (March 2020) and a period several months later (October 2020). We also sought to analyse which socio-demographic and psychosocial variables were more predictive of high levels of resilience in these healthcare professionals.

The results indicate that during the first period of confinement and restriction (W1 = March 2020), healthcare workers exhibited higher levels of resilience than in the following months (W2 = October 2020). Changes in the levels of depression were also significant, with higher levels in the second assessment period (W2), representing the "new normal". In contrast, levels of anxiety remained similar in both periods. This higher level of resilience in March (W1) may be due to a lack of awareness of the dramatic health consequences of COVID-19 on the population, as reported by other authors [49, 50]. Healthcare workers have struggled with the frontline, exposing themselves to the possibility of infection daily due to limited availability of protective equipment and increased workload. Additionally, increased levels of depression during the "new normal" (W2) can be seen as a psychological symptom which is a consequence of burnout suffered during the preceding months of the pandemic. These consequences also occur in other parts of the world, where health workers suffer high levels of depressive disorders, anxiety disorders, and severe posttraumatic stress disorder [51], informing that health systems in the world are not as effective as the face of global adversity. However, anxiety is the variable that most hinders the development of resilience in healthcare workers in continuous periods of highly demanding work, so it is important to promote psychological strategies to reduce anxiety in this population, especially those between one and six years or more than thirteen years of professional experience.

Regarding to the relationship between resilience and other variables, it is not surprising that protective variables (dispositional optimism, self-efficacy, and emotional intelligence) have a significant direct relationship with resilience, and have a significant inverse relationship with risk variables (depression and anxiety). These results are in line with previous research that has also assessed these relationships [12]. Self-efficacy is a variable that can promote higher levels of resilience in healthcare professionals, however this may depend on other contextual factors. Hence, in the second wave of the pandemic by COVID-19, the level of resilience is more related to dispositional optimism and positive expectations about the future (vaccination) as other studies have shown [52]. Therefore, self-efficacy appears to be a fundamental aspect in facing the beginnings of a pandemic, as it makes the individual aware that they have some control over adversity, following protective measures to avoid infection. A high level of self-efficacy increases the motivation to overcome challenging situations and thus respond in a resilient manner. These results are consistent with previous studies [53, 54]. Additionally, self-efficacy also enhances more problem-focused coping than emotion-focused coping, which promotes more efficient and effective responses to adverse situations as demonstrated in previous studies [55].

These results provide information about the clinical applicability of knowledge of protective variables, since, as previous research has shown, the COVID-19 pandemic has had a great impact on healthcare workers’ mental health. The strong relationship between self-efficacy and resilience is a consistent result in this research, proving to be a protective factor—along with emotional intelligence and dispositional optimism—that predicts resilience. This indicates the importance of promoting these protective factors to reduce the emotional effects of an adverse event, such as a pandemic, which requires continued professional effort. Resilience is an outcome that can be derived from the protective modulation of stress and burnout in healthcare professionals exposed to highly demanding work situations as demonstrated by previous studies [56, 57]. Psychological intervention with healthcare professionals is therefore important for reinforcing perceptions and feelings of confidence in one's own abilities (self-efficacy). This could be implemented in health centres and hospitals through individual or group psychological sessions to enhance the protective variables that predict resilient outcomes in extremely adverse situations. National healthcare policies about pandemic prevention must not neglect building resilience in their human capital.

The limitations of this research are related to the heterogeneity of the sample, although the inclusion of healthcare professionals with different occupations, working in both primary and specialised care, allowed for a more complete picture of the impact of the pandemic on that group. Finally, the assessment of all sociodemographic and psychosocial variables was done using self-report instruments that were not confirmed by external assessments. Despite that, this study provides evidence that sociodemographic and protective variables are more predictive of resilient responses to adverse situations such as current or future pandemics.

## Conclusion

The present study confirmed that certain protective factors in healthcare workers promote resilient outcomes and better adaptation to high stress and burnout situations. Nurses had high levels of anxiety and depression during the two waves of the COVID-19 pandemic, but this does not mean that protective factors could not be strengthened to enhance their level of positive adaptation in changing and demanding work contexts. Public health policy makers should promote effective prevention tools and continue to pay attention to the psychological state of nurses and related risk factors in order to intervene on protective aspects that promote greater stabilisation of the nursing team, and can prevent and reduce the risk of illness.

## Data Availability

Data available on request due to privacy/ethical restrictions. The data that support the findings of this study are available on request from the corresponding author. The data are not publicly available due to privacy or ethical restrictions.
